# Comparison of degenerative lumbar spondylolisthesis and isthmic lumbar spondylolisthesis: effect of pedicle screw placement on proximal facet invasion in surgical treatment

**DOI:** 10.1186/s12891-021-04962-7

**Published:** 2022-01-03

**Authors:** Peng Tao Wang, Jia Nan Zhang, Tuan Jiang Liu, Jun Song Yang, Ding Jun Hao

**Affiliations:** Spine Surgery, Honghui Hospital Affiliated, Xi’an Jiaotong University, Xi’an City, 710054 Shaanxi Province China

**Keywords:** degenerative lumbar spondylolisthesis, isthmus spondylolisthesis, pedicle screw insertion, intrusive articular process

## Abstract

**Background:**

Pedicle screw invasion of the proximal articular process will cause local articular process degeneration and acceleration, which is an important factor affecting adjacent segment degeneration. Although lumbar spondylolisthesis is a risk factor for screw invasion of the proximal joint, there is no clear conclusion regarding the two different types of spondylolisthesis. Therefore, the purpose of this study was to explore the influence of pedicle screw placement on proximal facet invasion in the treatment of degenerative spondylolisthesis and isthmic spondylolisthesis.

**Methods:**

In total, 468 cases of lumbar spondylolisthesis treated by decompression and fusion in our hospital from January 2017 to January 2020 were included in this retrospective study. Among them, 238 cases were degenerative spondylolisthesis (group A), and 230 cases were isthmic spondylolisthesis (group B). Sex, age, body mass index, bone mineral density, preoperative visual analog scale (VAS) and Oswestry Disability Index (ODI) scores, postoperative VAS and ODI scores at 1 month and 3 months, and angle of the proximal facet joint at the last follow-up were recorded and compared between the two groups. The degree of pedicle screw invasion of the proximal facet joint was graded and compared by the SEO grading method.

**Results:**

There were no significant differences in sex, age, body mass index, bone mineral density, preoperative VAS and ODI scores, or proximal facet joint angle between the two groups (P > 0.05). There was no significant difference in VAS and ODI scores between the two groups at 1 month and 3 months after the operation (P > 0.05). The VAS score of group A at the last follow-up was 1 (1,2). The VAS score of group B at the last follow-up was 3 (1,3). The ODI score of group A at the last follow-up was 6(4,26). The ODI score of group B at the last follow-up was 15(8,36). The VAS and ODI scores of the two groups at the last follow-up were significantly different (P < 0.05). According to the SEO grading method, the invasion of the proximal articular process by pedicle screw placement in group A involved 320 cases in grade 0, 128 cases in grade I and 28 cases in grade II. In group B, there were 116 cases in grade 0, 248 cases in grade I and 96 cases in grade II, with a significant difference (P < 0.01).

**Conclusion:**

In summary, a certain number of cases involving screws invading the proximal facet joint occurred in the two different types of lumbar spondylolisthesis, but the number in the isthmic spondylolisthesis group was significantly higher than that in the degenerative spondylolisthesis group, which caused more trauma to the proximal facet joint and significantly affected the patient prognosis.

**Supplementary Information:**

The online version contains supplementary material available at 10.1186/s12891-021-04962-7.

Lumbar spondylolisthesis is a common multiple-degenerative disease in the clinic. The incidence rate is 5%-7% [1], and the disease is most commonly seen in the lower lumbar spine. The clinical manifestations are lumbar pain, lower limb radiating pain, and neurogenic intermittent claudication. At present, the integrity of the isthmus of the vertebral arch can be divided into degenerative spondylolisthesis and isthmic spondylolisthesis. In the case of ineffective conservative treatment or obvious symptoms, surgical treatment is feasible [2–4]. At present, decompression + pedicle screw fixation + bone graft fusion are mostly used [5, 6]. Many mathematicians [7, 8] also believe that the addition of internal fixation can prevent postoperative spinal instability, increase the fusion rate and improve the reduction rate of the spondylolisthesis-affected vertebral body. Regarding the long-term effect, the stability of the spine with internal fixation is more obvious. However, the implantation of pedicle screws will invade the proximal facet joint to varying degrees. Previous literature has reported that lumbar spondylolisthesis is a risk factor for pedicle screw invasion of the proximal facet joint [9]. However, isthmic spondylolisthesis, due to the discontinuous bone defect in the isthmus, forms more scar tissue, hypertrophy of the ligamentum flavum, and multiple hyperplastic bones at the broken end of the isthmus. Therefore, it causes inaccurate positioning in the process of pedicle screw placement, and the pedicle screw easily invades the facet joint, thus causing damage to the normal proximal facet joint. The degeneration of the adjacent lumbar body and facet joints is further accelerated. A review of previous research reports revealed that there is no relevant literature on the impact of pedicle screw placement on proximal facet invasion in the surgical treatment of degenerative lumbar spondylolisthesis and isthmic lumbar spondylolisthesis. Therefore, we retrospectively analyzed the influence of pedicle screw placement on adjacent facet invasion in the treatment of degenerative lumbar spondylolisthesis and isthmic lumbar spondylolisthesis.

## Materials and methods

### Inclusion criteria

1. Lumbar spondylolisthesis was diagnosed according to the patient's symptoms, signs and imaging data; 2. Surgical segments were single segment; 3. Conservative treatment was ineffective for more than 6 months; 4. All patients had lower extremity neurological symptoms; 5. The operation was decompression reduction and fusion of pedicle screw fixation; 6. Manual placement of screws.

### Exclusion criteria

1. Severe osteoporosis; 2. Spinal tumor, metastasis, hemangioma and other neoplasms; 3. Metal allergy; 4. History of lumbar surgery; and 5. Congenital or traumatic spinal deformities; 6. Degenerative disease occurred in distal adjacent segments.

### Case data

The basic data of the two groups are shown in Table 1. In group A, there were 132 cases of L4 spondylolisthesis; 90 cases of L5 spondylolisthesis; 9 cases of L3 spondylolisthesis; and 7 cases of L2 spondylolisthesis (I ° slippage: 108 cases of spondylolisthesis; II ° slippage: 128 cases of spondylolisthesis; III ° slippage: 2 cases of spondylolisthesis). Group B had isthmic spondylolisthesis, including 116 cases of L4 spondylolisthesis; there were 103 cases of spondylolysis type L5 spondylolisthesis, 8 cases of L3 spondylolisthesis, and 3 cases of L2 spondylolisthesis (I ° slippage: 122 cases of spondylolisthesis; II ° slippage: 105 cases of spondylolisthesis; III ° slippage: 3 cases of spondylolisthesis). The last follow-up period: group A: (18.37±1.59 months); group B: (18.49±1.17 months).

**Table 1:** The basic data of the two groups

	GROUP A	GROUP B	P
SEX (MALE/FEMALE)	99/139	104/126	0.129
AGE (YEARS)	64.31±2.51	62.32±2.50	0.844
BMI (KG/M^2^)	26.30±1.75	25.41±1.90	0.446
BMD	-3.29±1.13	-3.39±1.04	0.151
ANGLE OF PROXIMAL FACET JOINT	32.24±1.82	31.90±2.01	0.085

### Therapeutic technique

After general anesthesia, the patient was placed in the prone position, disinfected according to the routine procedure of posterior lumbar surgery, covered with a sterile towel sheet, and pasted with knife edge film. The skin, subcutaneous tissue, and low back fascia were cut layer by layer, and the spinous process, lamina and bilateral facet of the vertebral body were fixed by blunt subperiosteal dissection in addition to the spinous process. According to the anatomical structure and the external position of the transverse process and lamina, with the aid of C-arm perspective, the entry point and trajectory are determined through the positioning needle. The anatomical mark is "herringbone ridge". If the "herringbone ridge" cannot be accurately located, the point 3 mm outside the intersection of the vertical extension line of the lateral edge of the isthmus and the horizontal line of the central axis of the transverse process are selected as the needle entry point. The spinous process, lamina and inferior articular process of spondylolisthesis were removed, and the thickened ligamentum flavum was removed. The dural sac and nerve root were pulled open, the annulus fibrosus was cut with a small sharp knife, and the intervertebral disc was treated with reamer, curette and nucleus pulposus forceps, resulting in bleeding of the upper and lower endplates. The nerve root canals were expanded after full decompression, and nerve root relaxation was explored after full decompression. The appropriate longitudinal connecting rod was prebent, the intervertebral space of the spondylolisthesis vertebrae was moderately expanded, and the reduction was lifted and expanded. After the wound was washed and the model was tested properly, the bone particles obtained by decompression during the operation were trimmed to a suitable size and then implanted in front of the intervertebral space. The intervertebral fusion cage filled with the treated autologous bone particles was implanted in the rear of the cage, approximately 5 mm away from the posterior edge of the vertebral body, and the intervertebral space was compressed and clamped with appropriate holding screws. The wound was rinsed with normal saline, and hemostasis was carefully observed; when no active bleeding was observed in the wound, a drainage tube was placed in the wound, the wound was sutured layer by layer, and a sterile dressing was used as a pressure dressing. This study was reviewed and approved by the ethics committee of Honghui Hospital Affiliated with Xi’an Jiaotong University.

**Fig. 1:**
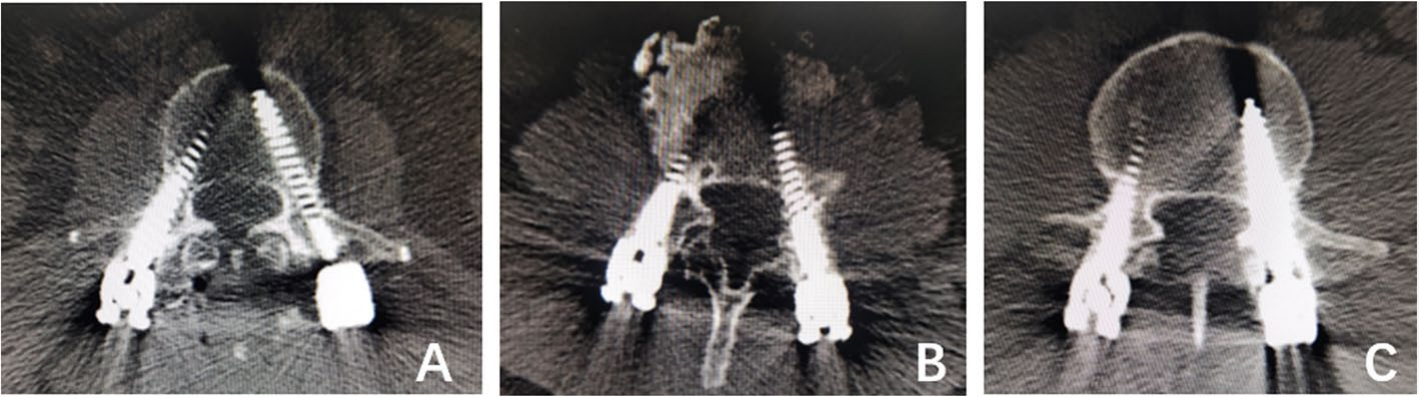
**A** The left and right pedicle screws did not invade the facet joint, grade 0. **B** The left screw contacted or was suspected to have invaded the joint, grade 1. The right pedicle screw did not invade the facet joint, grade 0. **C** The right screw contacted or was suspected to have invaded the joint, grade 1. The left screw clearly invaded the facet joint, grade 2

In this study, 562 patients received surgical treatment from different doctors in three medical groups in the same department. To eliminate the interference of human factors, the chief surgeons of the included cases were senior chief surgeons with more than 10 years of experience. According to the grading method of Seo et al. [10]: Grade 0 - the screw clearly avoids the joint; Grade 1 – the screw contacts or is suspected to have invaded the joint; Grade 2 - the screw clearly destroys the joint, and two senior surgeons evaluate whether the proximal facet joints of the lumbar spine are invaded through postoperative CT examination. If the views of the two doctors conflict, a third senior chief physician shall be used for evaluation and judgment to make a clear conclusion (Fig. 1).

1.5 Statistical methods: SPSS 19.0 software was used to analyze and process the data in this study. χ^2^ and T tests were used for the measurement data in accordance with the normal distribution, P < 0.05 showed that the difference was statistically significant; Z {m (Q1, Q3)} was used for the measurement data in the the nonparametric test, P < 0.05 showed that the difference was statistically significant. The analysis of variance (ANOVA test) was used for the data of multiple recombination, and LSD-t test was used for further pairwise comparison. The rank sum test was used to test the postoperative pedicle screw invasion of the facet joint.

## Results

There was no significant difference in the preoperative VAS and ODI scores or the VAS and ODI scores after 1 month and 3 months of follow-up between the two groups, but there was a significant difference in the last follow-up VAS and ODI scores between the two groups (Tables 2 and 3).

**Table 2:** Statistical analysis of VAS score before and after operation in the two groups

	Preoperative VAS score	The VAS score was followed up at 1 month	The VAS scores were followed up at 3 months	VAS score at the last follow-up	F	P
Group A	8(7, 8)	4(4, 5)	2(2, 3)	1(1, 2)	2754.643	<0.001
Group B	7(7, 8)	5(4, 5)	3(2, 3)	3(1, 3)	1620.675	<0.001
P	0.145	0.148	0.114	<0.001		
Z	-1.458	-1.446	-1.580	-8.630		

Note: VAS preoperative scores of the two groups were compared with the 1-month, 3-month and last follow-up VAS scores. P value <0.001.

**Table 3:** Statistical analysis of ODI score before and after operation in the two groups

	Preoperative ODI score	The ODI score was followed up at 1 month	The ODI scores were followed up at 3 months	ODI score at the last follow-up	F	P
Group A	68(63.5, 74)	56(52, 58)	30(26, 36)	6(4, 26)	1683.740	<0.001
Group B	68(63, 76)	56(50, 60)	28(26, 36)	15(8, 36)	1124.044	<0.001
P	0.724	0.848	0.642	<0.001		
Z	-0.354	-0.192	-0.465	-8.413		

Note: ODI preoperative scores of the two groups were compared with those of the 1-month, 3-month and last follow-up ODIs. P value <0.001.

2.2 There were differences in the number and grade of cases of postoperative pedicle screw invasion of facet joints between the two groups. In group A, there were 320 cases of grade 0, 128 cases of grade I and 28 cases of grade II. In group B, there were 116 cases of grade 0, 248 cases of grade I and 96 cases of grade II (Table 4).

**Table 4:** Statistical analysis of the two groups of patients with different degrees of proximal facet joint invasion

The grade of invasion of facet joint by a pedicle screw	Group A (Number of cases)	Group B (Number of cases)	P	Z
Grade 0	320	116	<0.001	-9.050
Grade 1	128	248		
Grade 2	28	96		

**Note:** The level and number of cases of pedicle screws in different degrees were compared between the two groups**.** P value <0.001.

## Discussion

**Fig. 2:**
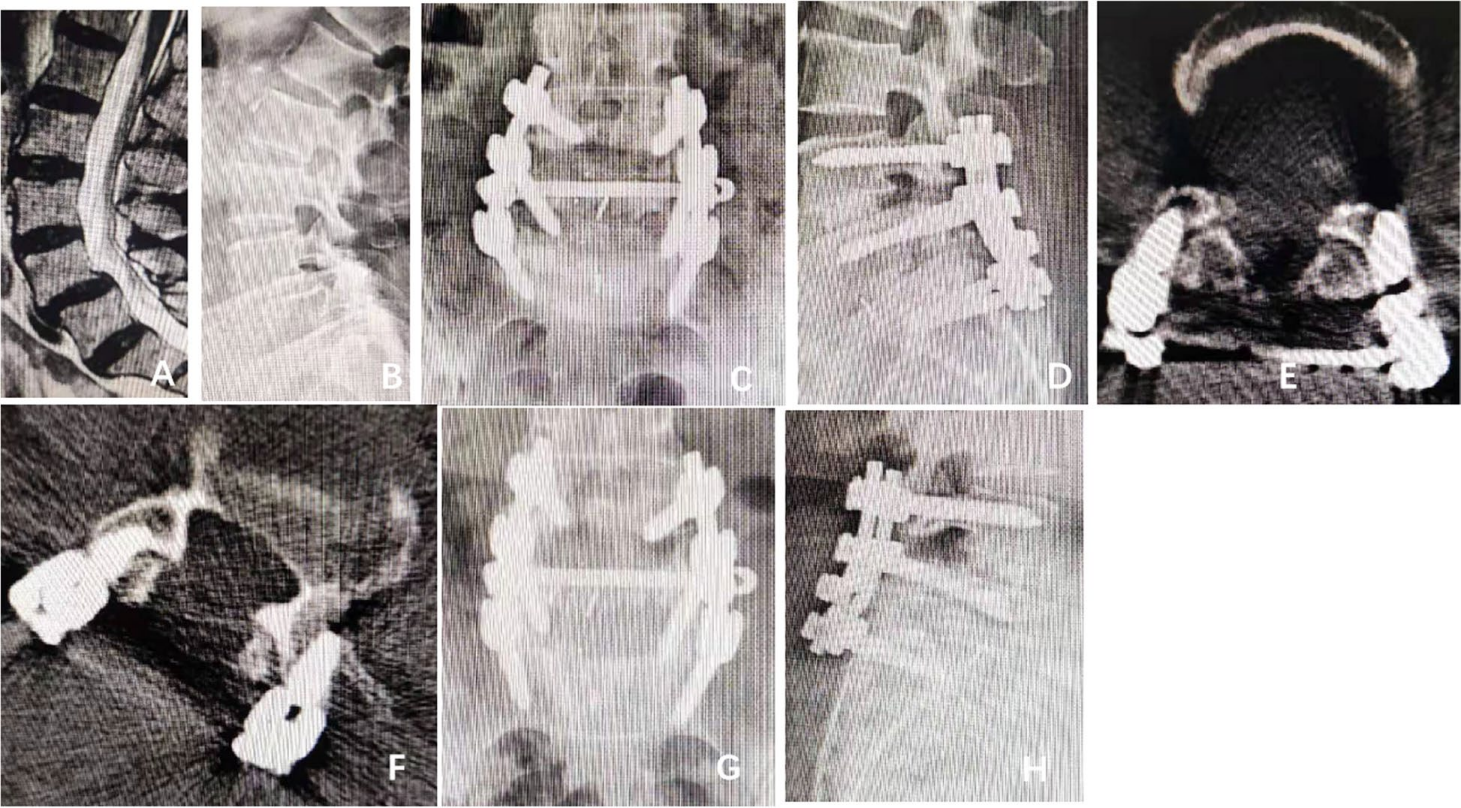
A 68-year-old female was diagnosed with degenerative lumbar spondylolisthesis. She underwent posterior decompression, bone graft fusion and internal fixation in our hospital. Figures **A** and **B** show that the patient had spondylolisthesis of L4 (i°). The isthmus was intact. Figures **C** and **D** show the position of internal fixation after the operation. Figure **E** shows that the left pedicle screw of the L4 vertebral body invaded the proximal articular process after the operation. According to the SEO grading method, the patient can be classified into grade 2, and the pedicle screw obviously breaks the ring joint. According to the SEO classification, the patient can also be classified into grade 0. As shown in Figure F, the patient was reexamined at 2 months after the operation, and CT reconstruction showed that the left pedicle screw of the L4 vertebral body invaded the proximal articular process. According to the SEO grading method, the patient can be classified into two grades. According to the SEO classification, the patient can be classified into grade 0. Figures G and H show the position of internal fixation at 2 months after the operation

**Fig. 3:**
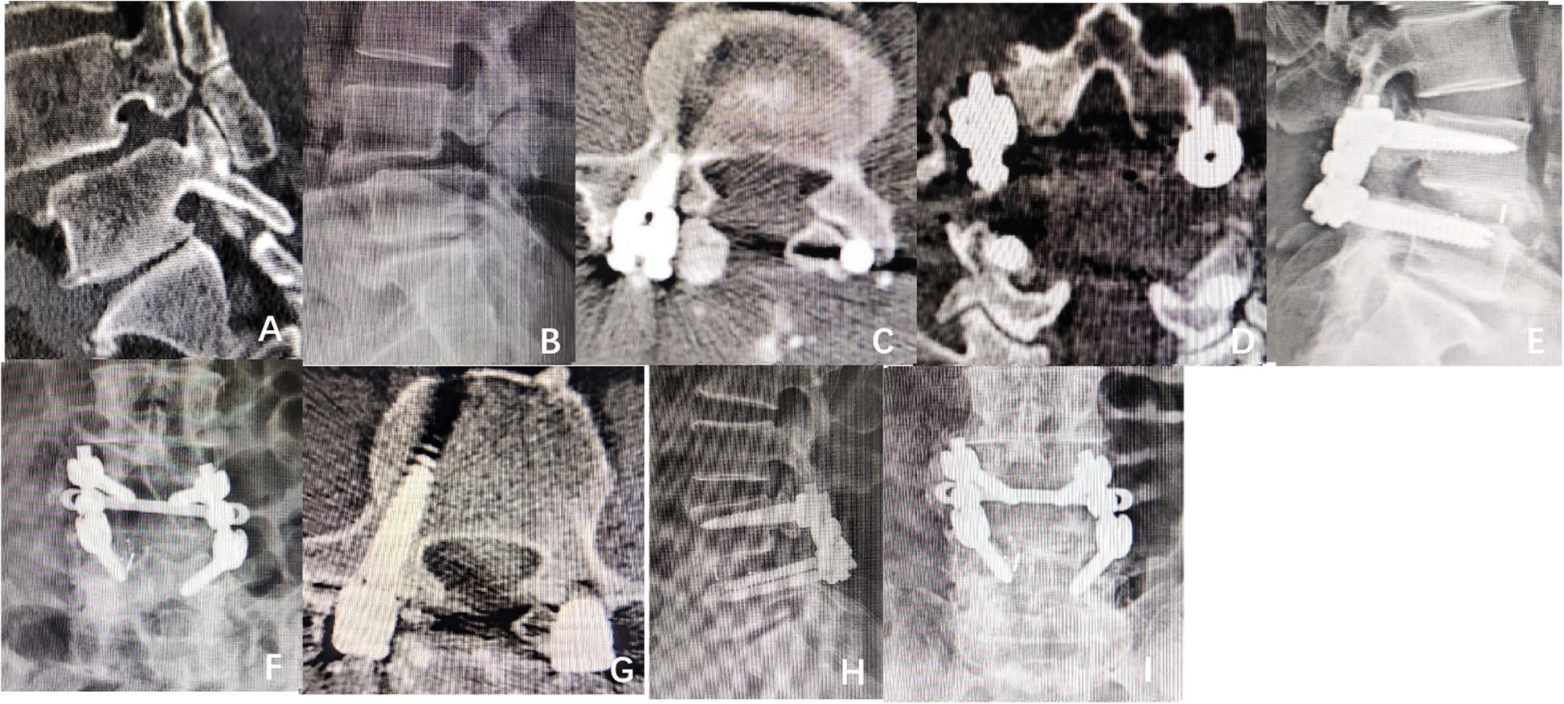
A 61-year-old male was diagnosed with isthmic spondylolisthesis. He underwent posterior decompression, bone graft fusion and internal fixation in our hospital. Figures **A** and **B** show that the patient had spondylolisthesis of L4 (i°). The isthmus of the bilateral pedicle of the L4 vertebral body was not connected. Figures **C** and **D** show that the pedicle screws on both sides of the L4 vertebral body invaded the proximal articular process after the operation. According to the SEO grading method, the patient can be classified into grade 2, and the pedicle screws obviously break the ring joint. Figures **E** and **F** show the position of internal fixation after the operation. As shown in Figure **G**, the patient was reexamined 2 months after the operation, and the pedicle screws of both sides of L4 invaded the proximal articular process. According to the SEO grading method, the patient can be classified into two grades. Figures **H** and **I** show the position of internal fixation at 2 months after the operation

**Fig. 4:**
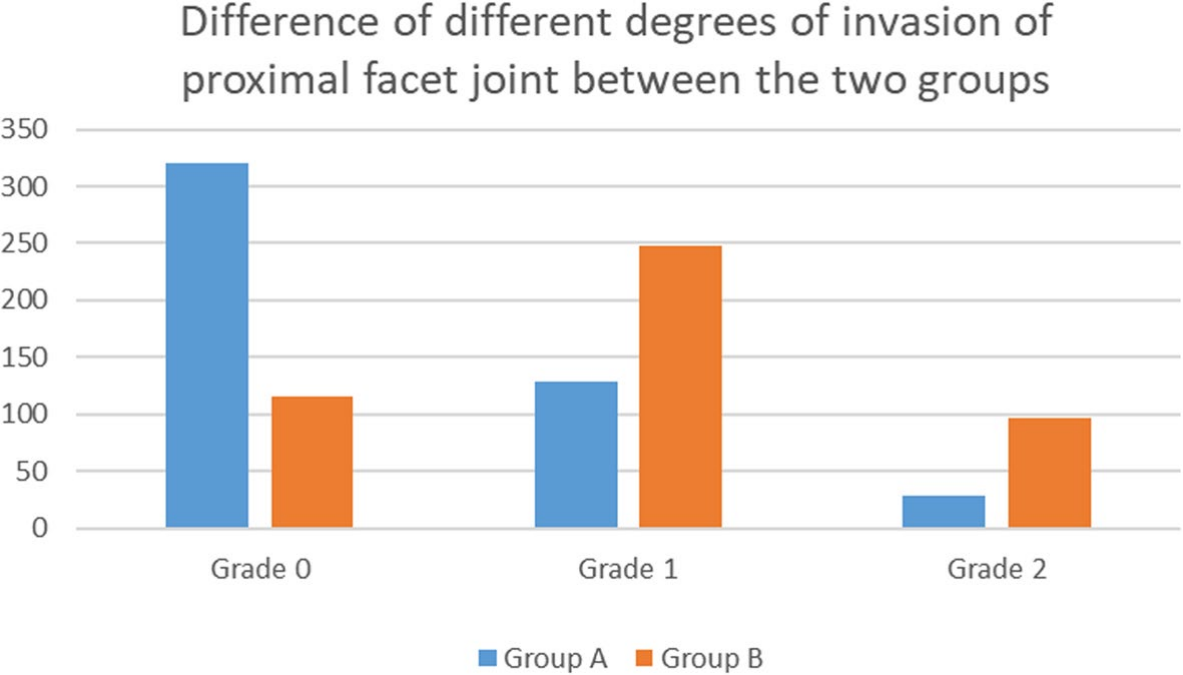
The difference between degenerative lumbar spondylolisthesis and isthmic lumbar spondylolisthesis

Lumbar spondylolisthesis is a common clinical disease in which an upper vertebral body slides relative to the adjacent lower vertebral body, resulting in a series of clinical symptoms, such as low back pain and corresponding neurological symptoms. At present, the clinical treatment of lumbar spondylolisthesis can be divided into conservative treatment and surgical treatment. Generally, patients with mild symptoms and lower extent of disease can choose conservative treatment. Surgical treatment is suitable for failed conservative treatment with severe symptoms that significantly affect the quality of life; such operative therapy involves decompression + reduction + bone graft fusion + internal fixation as the principle [11]. At present, the pedicle screw system is widely used in internal fixation of lumbar spondylolisthesis [12]. However, Matsuzaki et al. [13] reported for the first time that pedicle screws can invade the proximal facet joint and cause trauma to the facet joint after lumbar surgery. Shah et al. [14] first used CT reconstruction to observe the distance between the pedicle screw and proximal facet joint and defined the injury of the pedicle screw and connecting rod to the proximal facet joint. Relevant literature has reported that a degree of lumbar spondylolisthesis > 10% is an independent risk factor for proximal facet joint injury, and the incidence of adjacent facet joint invasion is higher after pedicle screw implantation [9]. However, there is no relevant literature reporting the impact of pedicle screw placement on proximal facet invasion in the surgical treatment of degenerative lumbar spondylolisthesis and isthmic lumbar spondylolisthesis. According to the grading method of Seo et al. [10], the degree of postoperative pedicle screw invasion of the proximal articular process in the two groups was graded. The results of this study showed that there were 320 cases of grade 0, 128 cases of grade I, and 28 cases of grade II (Group A, Fig. 2). There were 116 cases of grade 0, 248 cases of grade I and 96 cases of grade II (Group B, Fig. 3). Therefore, we found that both groups of pedicle screws had the possibility of invading the proximal facet joint. Previously, Sears et al. [15] reported that pedicle screw placement invaded the proximal facet joint and easily caused damage to this joint. Park et al. [16] reported that intraoperative pedicle screw implantation is the main factor of proximal facet joint injury. Related literature [17, 18] has reported that common pedicle screw implantation techniques, such as percutaneous pedicle screw fixation and open pedicle screw fixation, may cause proximal facet joint invasion and proximal facet joint trauma. Chung et al. [19] and He et al. [20] reported that different positions of screw placement during surgery will have different effects on the invasion of the proximal facet joint. Therefore, the positioning accuracy of the screw entry point during the operation can affect the angle and depth after pedicle screw implantation. When the angle and depth after pedicle screw implantation are affected, it easily invades the proximal facet joint. Therefore, when lumbar degenerative changes occur, spondylolisthesis results in increased physiological flexion of lumbar spine. The operative field of vision is deep when the pedicle screw is implanted. Local structural disorder of the spondylolisthesis vertebral body [9] poses many difficulties for pedicle screw implantation, such as inaccurate positioning, angle deviation of the screw implantation, and so on. It is easy to position the pedicle screw in a way that causes it to invade the proximal facet joint. However, isthmic spondylolisthesis is more likely due to pedicle fracture. With hypertrophy of vertebral facet, hyperplasia is obvious and leads to many osteophytes in the isthmus and scar tissue formation. Moreover, because of the degeneration of the facet joint itself, the facet joint is hardened and deformed. With the formation of pseudarthrosis [21], the normal anatomical structure and landmarks are changed. This has a more significant impact on the positioning of the pedicle screw, greatly affects the correct angle and depth of the pedicle screw after the operation, and is more likely to damage the proximal facet joint and invade it. Therefore, we can conclude that the local structure and anatomical landmarks of isthmic spondylolisthesis are changed, and the influence of pedicle screw implantation is more obvious. To protect the joint capsule of the adjacent articular process, the position of the joint capsule and screw placement point is not clear enough during reduction and decompression. To sum up, firstly, at present, when we perform surgical treatment for lumbar spondylolisthesis, we usually perform reduction, which will lead to the height difference after pedicle screw implantation on both sides. Therefore, a few patients have too deep pedicle screw implantation. Second, when the vertebral isthmus breaks, scar tissue is formed locally and the anatomical marks are blurred, which affects the placement of nails. Finally, in order to protect the facet joint, it is not exposed enough. It is more likely that the proximal facet joint will be invaded by the pedicle screw after the operation (Fig. 4). In this study, intraoperative pedicle screw implantation was performed by senior surgeons using unarmed implantation. At present, domestic lumbar spine surgery in the process of pedicle screw implantation involves more unarmed implantation, and unarmed implantation of pedicle screws can possibly result in invasion of the proximal facet joint. Sakaura [22] reported that cortical bone trajectory screw technology has different degrees of invasion into the proximal facet joint, but compared with posterior lumbar interbody fusion (PLIF) traditional pedicle screw fixation, the possibility of cortical bone trajectory screw technology invasion to the proximal facet joint will be reduced. Yson [23] reported the application of three-dimensional CT navigation-assisted pedicle screw implantation. The results showed that the incidence of invasion of the proximal facet joint by pedicle screws was still high. Therefore, we found that the proximal facet joint will be invaded by either manual screw implantation or pedicle screw implantation with new technology and new auxiliary devices. However, according to relevant literature reports [24, 25], new auxiliary equipment or instruments can reduce the invasion of pedicle screws to adjacent facet joints. However, this technique has not been widely used in clinical practice, and there is no big-data or large-sample-size support. At present, this approach is still controversial with no clear conclusion.

**Fig. 5:**
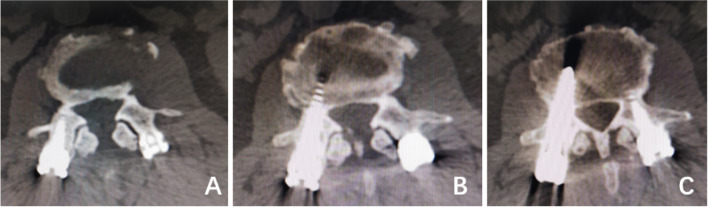
A 74-year-old male was diagnosed with isthmic spondylolisthesis. **A**, **B** and **C**. During the follow-up and reexamination 15 months after the operation, according to the SEO grading method, CT showed that the left pedicle screw had grade 1 invasion to the facet joint, there was an air sign in the left proximal facet joint space, there was local facet hyperplasia and degeneration, and traumatic arthritis was visible

According to the results of the study, there was no significant difference in VAS and ODI scores or proximal facet joint angles between the two groups in the short term after surgery, and there was a significant difference in VAS and ODI scores between the two groups at the last follow-up. The author believes that, in the short period after surgery, the patients are in the recovery stage, the times of ambulation are fewer, and the duration is short; therefore, the impact of pedicle screw invasion of the facet joint is lower, and there is less damage to the facet joint. In related research reports, when the lumbar spine flexes, extends and rotates, it will stretch and twist the torque. Therefore, when the pedicle screw is close to the facet joint, it is easier for the pedicle screw to conduct a stress effect on the facet joint, rendering the joint capsule vulnerable to damage. At the same time, the pressure of the intervertebral space and the contact force of the facet joint increase significantly, which causes damage to the facet joint. Cardoso et al. [26] also reported that, after the pedicle screw invaded the facet joint, it adversely affects the adjacent proximal segments by increasing the contact force of the facet joint and the pressure of the intervertebral disc, thus accelerating degeneration of the facet joint [27, 28]. Therefore, patients complain of low back pain in varying degrees during follow-up, and a few patients even complain of mild neurological symptoms of both lower limbs (Fig. 5). As a result, there was no significant difference in VAS and ODI scores between the two groups in the short term after the operation. However, in the long term, after an extensive period of recovery, the number and time of patients getting out of bed gradually increased, and the impact of pedicle screw invasion of the facet joint gradually became prominent. After a long period, the trauma to the corresponding facet joint was more apparent; at the same time, we believe that the greater the degree of pedicle screw invasion of the facet joint, the greater the impact on the facet joint. For example, the impact and destruction degree of grade 2 invasion on the facet joint is greater than that of grade 1 invasion, and patients may have low back pain and neurological symptoms of both lower limbs, which may affect the VAS and ODI scores at the last follow-up. The VAS and ODI scores at the last follow-up were significantly higher than those in patients without pedicle screw invasion of the facet joint, and thus, the VAS and ODI scores of the two groups at the last follow-up were significantly different. In 1987, the concept of minimum clinical importance difference (MCID) was first proposed. Canadian scholars then defined it and considered MCID as the change value of the minimum questionnaire dimension score recognized by patients without considering side effects and costs. Therefore, in relevant clinical studies, we obtained P < 0.05, and the difference was greater than MCID. The differences between the two groups are both statistically significant and clinically significant. Therefore, according to the evaluation indexes proposed by Gum et al. [29], compared with the results of this study, the VAS and ODI scores of low back pain are of clinical significance. However, there is still no gold standard for the formulation of MCID; thus, the commonly used scores of spinal surgery, such as VAS score, ODI score, JOA score, and NDI score, need to be further studied. After lumbar fusion and internal fixation, because the pedicle screw is disturbed in the facet joint, damage to the facet joint will accelerate the degeneration of the facet joint, further affecting the degeneration of adjacent segments of the fused vertebral body and the occurrence of degenerative diseases of adjacent segments as well as reducing the stability of the spine [26]. Through the comparisons in this study, we found that pedicle screw implantation in the treatment of isthmic spondylolisthesis is more likely to invade the adjacent facet joints, accelerate the degeneration of adjacent facet joints, and then affect the adjacent segment of vertebral degeneration. Finally, this is a single-center retrospective study that has some limitations and needs to be further verified by a large sample of multicenter prospective studies.

## Supplementary Information

**Additional file 1.**

**Additional file 2.**

## Data Availability

All data and materials in this study are clinical data, which are available. All data generated or analyzed during this study are included in this published article [and its supplementary information files].
